# BNIP3L/BNIP3‐Mediated Mitophagy Contributes to the Maintenance of Ovarian Cancer Stem Cells

**DOI:** 10.1111/jcmm.70704

**Published:** 2025-10-13

**Authors:** Na Li, Tejinder Pal khaket, Yajing Yang, Linzhou Wang, Shurui Cai, Aidan Li, Elsa Wani, Jessica Miao, Nan Zhang, Qingfei Zheng, Junran Zhang, Xuefeng Liu, Selvendiran Karuppaiyah, Dehua Pei, Qi‐En Wang

**Affiliations:** ^1^ Department of Radiation Oncology, Comprehensive Cancer Center The Ohio State University Columbus Ohio USA; ^2^ Department of Pathology, College of Medicine The Ohio State University Columbus Ohio USA; ^3^ Department of Obstetrics and Gynecology, College of Medicine The Ohio State University Columbus Ohio USA; ^4^ Department of Chemistry and Biochemistry, College of Biology The Ohio State University Columbus Ohio USA

**Keywords:** BNIP3, BNIP3L, cancer stem cells, DNA‐PK, mitophagy, NF‐κB, ovarian cancer

## Abstract

Ovarian cancer remains the most lethal gynaecological malignancy, with tumour recurrence and chemoresistance posing significant therapeutic challenges. Emerging evidence suggests that cancer stem cells (CSCs), a rare subpopulation within tumours with self‐renewal and differentiation capacities, contribute to these hurdles. Therefore, elucidating the mechanisms that sustain CSCs is critical for improving treatment strategies. Mitophagy, a selective process for eliminating damaged mitochondria, plays a key role in maintaining cellular homeostasis, including CSC survival. Our study demonstrates that ovarian CSCs exhibit enhanced mitophagy, accompanied by elevated expression of the mitochondrial outer membrane receptors BNIP3 and BNIP3L. Knockdown of BNIP3 or BNIP3L significantly reduces mitophagy and impairs CSC self‐renewal, indicating that receptor‐mediated mitophagy is essential for CSC maintenance. Mechanistically, we identify that hyperactivated NF‐κB signalling drives the upregulation of BNIP3 and BNIP3L in ovarian CSCs. Inhibition of NF‐κB signalling, either via p65 knockdown or pharmacological inhibitors, effectively suppresses mitophagy. Furthermore, we demonstrate that elevated DNA‐PK expression contributes to the constitutive activation of NF‐κB signalling, thereby promoting mitophagy in ovarian CSCs. In summary, our findings establish that BNIP3/BNIP3L‐mediated mitophagy, driven by DNA‐PK‐dependent NF‐κB hyperactivation, is essential for CSC maintenance. Targeting the DNA‐PK/NF‐κB/BNIP3L‐BNIP3 axis to disrupt mitochondrial quality control in CSCs represents a promising therapeutic strategy to prevent ovarian cancer recurrence and metastasis.

**Abbreviations**
ALDH: Aldehyde dehydrogenase
Baf A1: Bafilomycin A1
BNIP3: BCL2 Interacting Protein 3
BNIP3L: BCL2 Interacting Protein 3 Like
ChIP: Chromatin immunoprecipitation
CSCs: Cancer stem cells
DNA‐PKcs: DNA‐dependent protein kinase catalytic subunit
EOC: Epithelial ovarian cancer
FUNDC1: FUN14 Domain Containing 1
GSEA: Gene Set Enrichment Analysis
HGSOC: high‐grade serous ovarian carcinoma
HSCs: Haematopoietic stem cells
iPSCs: induced pluripotent stem cells
LIRs: LC3‐interacting regions
NIX: BCL2 Interacting Protein 3 Like
OMM: Outer mitochondrial membrane
PINK1: PTEN‐induced kinase 1

## 1 Introduction

Epithelial ovarian cancer (EOC), particularly high‐grade serous ovarian carcinoma (HGSOC), is the most lethal malignancy of the female reproductive system, with a 5‐year survival rate of only 30% in advanced stages [1]. Despite initial responsiveness to conventional chemotherapy following surgical cytoreduction, more than 70% of EOC patients experience tumour metastasis and recurrence, ultimately leading to poor prognosis and limited treatment options. The median survival for patients with recurrent ovarian cancer is less than 2 years, underscoring the urgent need to address tumour recurrence and therapeutic resistance.

A critical challenge in EOC treatment is the presence of a subpopulation of cancer stem cells (CSCs), which possess heightened tumorigenic potential and resistance to chemotherapy. These CSCs are implicated in tumour recurrence and treatment failure, ultimately driving disease progression [2, 3, 4]. CSCs possess characteristics of normal stem cells, particularly the ability of self‐renewal and differentiation, which are essential for their role in tumour initiation, progression, metastasis, and therapy resistance. The stemness properties and survival of CSCs depend not only on multiple signalling pathways but also on metabolic adaptations. However, whether CSCs primarily rely on glycolysis or mitochondrial metabolism as their principal energy source remains controversial. Emerging evidence suggests that CSCs exhibit metabolic plasticity, allowing them to dynamically switch between glycolysis and oxidative phosphorylation (OXPHOS) in response to microenvironmental changes [5]. This adaptability enables CSCs to survive under metabolic stress, evade therapeutic targeting, and sustain tumour progression [5, 6]. Therefore, elucidating mitochondrial dynamics in CSCs is crucial for understanding the mechanisms that sustain CSC survival and stemness, and providing new therapeutic opportunities to disrupt CSC maintenance and improve treatment outcomes.

Mitochondrial homeostasis is tightly regulated through biogenesis, degradation, fission, and fusion. Among these processes, selective autophagy‐mediated mitochondrial degradation, known as mitophagy, has recently emerged as a key regulator of cancer stemness [7, 8, 9, 10] and has garnered significant interest as a potential target for anticancer therapy. Mitophagy is a highly selective form of autophagy that maintains mitochondrial quality by eliminating damaged or dysfunctional mitochondria [11]. Mitophagy is crucial for sustaining metabolic homeostasis, preserving energy production, and mitigating oxidative stress, thereby enhancing their survival, resistance to apoptosis, and adaptability to the tumour microenvironments.

Mitophagy primarily occurs through two key pathways: receptor‐mediated mitophagy and ubiquitin‐dependent mitophagy. In receptor‐mediated mitophagy, specific mitochondrial receptor proteins, such as BCL2 Interacting Protein 3 (BNIP3), BCL2 Interacting Protein 3 Like (BNIP3L, or NIX) and FUN14 Domain Containing 1 (FUNDC1), are translocated and stabilised on the outer mitochondrial membrane (OMM), interact with LC3 (a protein involved in autophagosome formation) through LC3‐interacting regions (LIRs) to recruit autophagosomes and facilitate mitochondrial degradation [12]. Although in ubiquitin‐dependent mitophagy, PTEN‐induced kinase 1 (PINK1) accumulates on the OMM, leading to the recruitment, stabilisation, and activation of Parkin, an E3 ubiquitin ligase, which ubiquitinates OMM proteins, signalling the autophagy machinery to engulf and degrade the mitochondria. Both pathways ensure cellular homeostasis by eliminating dysfunctional mitochondria and preventing oxidative stress‐induced damage.

The NF‐κB signalling pathway, which has been found to be constitutively activated in CSCs from a variety of cancers, participates in the maintenance, expansion, proliferation, and survival of CSCs in breast tumour [12, 13, 14, 15], glioblastoma [16], pancreatic cancer [17], colorectal cancer [18], and ovarian cancer [19, 20, 21]. NF‐κB typically exists in the cytoplasm as an inactive complex bound to inhibitor proteins (IκBs). Upon activation by various stimuli (e.g., cytokines, stress, infection, or oncogenic signals), the IκB kinase (IKK) complex phosphorylates IκB proteins, leading to their degradation via the ubiquitin‐proteasome system. This allows NF‐κB dimers, often composed of p65 (RelA) and p50, to translocate into the nucleus, where they regulate the expression of genes [22]. Previous studies have demonstrated that NF‐κB regulates mitophagy by upregulating the expression of the p62/SQSTM1 adaptor molecule in macrophage [23]. Additionally, the nonclassical NF‐κB pathway, involving IκB kinase‐α (IKK‐α), has been implicated in modulating mitochondrial dynamics and morphology by regulating the Optic atrophy 1 protein (OPA1) [24]. However, the regulation of mitophagy by the NF‐κB signalling in cancer cells, particularly in CSCs, remains unclear.

In this study, we demonstrated that ovarian CSCs exhibit elevated levels of mitophagy, primarily mediated by the upregulation of BNIP3L and BNIP3, both of which play crucial roles in maintaining their self‐renewal ability. Mechanistically, we identified that hyperactivated NF‐κB signalling, induced by the overexpression of DNA‐dependent protein kinase (DNA‐PK), enhances BNIP3L/BNIP3 expression, leading to increased mitophagy in ovarian CSCs.

## 2 Materials and Methods

### 2.1 Cell Culture

The human EOC cell lines including PEO1, OVCAR3, OVCAR4, and Kuramochi have been described previously [25]. All cell lines were authenticated via Short Tandem Repeat (STR) DNA profiling and routinely tested for mycoplasma contamination. To enrich for CSCs, these EOC cells were cultured in serum‐free 3D Tumour Sphere Medium XF (PromoCell, Heidelberg, Germany) in ultra‐low attachment dishes (Corning, Glendale, AZ) for at least 12 days.

### 2.2 Inhibitors, Plasmids, shRNA, Gene Transfection, and Lentivirus Packaging

NF‐κB selective inhibitors BAY 11–7082 and JSH‐23, along with DNA‐PK inhibitors KU‐0060648 and AZD‐7648, were purchased from MedChemExpress (Monmouth Junction, NJ). Plasmids, siRNAs, and shRNAs used in this study are listed in Table S1. Adherent cells were transfected with plasmids, siRNAs, and shRNAs using Lipofectamine 2000 reagent (ThermoFisher Scientific, Waltham, MA) according to the manufacturer's protocol, whereas spheroid cells were infected with lentivirus‐packaged shRNA.

### 2.3 Establishment of Cumate‐Inducible Stable Cell Lines

IκBαSR cDNA was subcloned from pcDNA‐IκBαSR (a gift from Dr. Denis Guttridge) into the pCDH‐CuO‐MCS SparQ vector (SBI, Palo Alto CA) to generate the SparQ‐IκBαSR plasmid. OVCAR3 cells were co‐transduced with SparQ‐IκBαSR and the CymR repressor plasmid (SBI). GFP‐positive cells were isolated by fluorescence‐activated cell sorting (FACS) and stable single‐cell clones of stably were validated by immunoblotting after cumate induction.

### 2.4 NF‐κB‐Reporter Assay

OVCAR3 cells were transfected with the pHAGE NFkB‐TA‐LUC‐UBC‐GFP‐W plasmid (Addgene, Watertown, MA). GFP‐positive cells were isolated via FACS to generate stable OVCAR3‐NF‐κB‐Luc reporter cells. These cells were seeded in 96‐well plates and treated with inhibitors for 48 h. After treatment, cells were washed with PBS, and luciferase activity was quantified using the Luciferase Assay System (Promega, Madison, WI) according to the manufacturer's instructions, with readings obtained on a GloMax Discover Microplate Reader (Promega).

### 2.5 Confocal Microscopy

OVCAR3 cells were stably transfected with pmRFP‐LC3 plasmids and selected using neomycin. Adherent and spheroid cultured cells were seeded into 35‐mm dishes and then treated with Baf A1 (100 nM) for 18 h. Cells were then stained with Hoechst 33342 (Thermo Fisher Scientific) to label nuclei and with MitoTracker Green (ThermoFisher Scientific) to label mitochondria. Multichannel stacks were sequentially recorded using a Zeiss LSM510 Meta confocal microscope (Zeiss, Oberkochen, Germany) with a 63 × oil immersion objective. Image analysis was performed using ImageJ (NIH).

### 2.6 Flow Cytometry Analysis and Cell Sorting

Mitochondria mass was assessed by staining cells with MitoTracker Green (ThermoFisher Scientific) and analysing them with a BD LSR II flow cytometer. ALDH activity analysis and cell sorting were performed with the ALDEFLUOR kit (STEMCELL Technologies, Cambridge, MA) using BD LSR II or BD Aria III flow cytometers, as previously described [26]. CD44^+^CD117^+^ cell sorting was conducted using PE‐conjugated CD117 and FITC‐conjugated CD44 antibodies (BD Pharmingen, San Diego, CA), followed by flow cytometry on a BD Aria III, as described [26].

### 2.7 Quantitative Real‐Time PCR

Total RNA was extracted from cells using the TRIzol reagent (ThermoFisher Scientific). First‐strand cDNA was synthesised using the reverse transcription system (ThermoFisher Scientific) in a 20 μL reaction containing 1.5 μg total RNA. The total cDNA was diluted to 60 μL with DEPC water, and a 2.5 μL aliquot of cDNA was amplified using Fast SYBR Green PCR Master Mix (Thermo Fisher Scientific) on a Quant Studio 3 system (ThermoFisher Scientific). Expression levels were normalised to 18S expression. Primer sequences are listed in Table S2.

### 2.8 Cellular Fractionation

For nuclei and cytoplasm separation, cells were suspended in hypotonic buffer (10 mM HEPES pH 7.9, 10 mM KCl, 10 mM MgCl₂, 1 mM PMSF) and incubated on ice for 10–15 min. Triton X‐100 (0.1%) was then added, and the suspension was rotated at 4°C for 10 min, followed by centrifugation at 1500 × *g* for 5 min at 4°C to obtain the pellet. The supernatant was clarified by high‐speed centrifugation (18,000 × *g*, 15 min, 4°C) to isolate the cytoplasmic fraction. The pellet was washed with 20 μL STM buffer (50 mM Tris–HCl pH 7.4, 0.25 M sucrose, 5 mM MgCl_2_, 1 mM PMSF) to isolate the nuclear fraction. The nuclear and cytoplasmic fractions were subsequently used for western blot analysis. For mitochondrial isolation, cells were treated with Baf A1 (100 nM) for 18 h. Mitochondrial fractionation was then performed using a mitochondrial isolation kit (MITOISO2, Millipore Sigma, St. Louis, MO) according to the manufacturer's protocol.

### 2.9 Western Blot Analysis

Whole cell lysates were prepared using the SDS lysis buffer and Western blotting was conducted as described previously [27]. The primary antibodies used in this study are listed in Table S3.

### 2.10 Sphere Formation Assay

The sphere‐forming ability of ovarian cancer cells were assessed using a semi‐solid sphere formation assay as previously described [26]. Meanwhile, 100 cells per well were seeded in a standard six‐well plate and cultured in RPMI 1640 medium supplemented with 10% FBS to evaluate colony formation rate. Tumour spheres were counted, and the sphere formation rate was normalised to the colony formation rate.

### 2.11 Chromatin Immunoprecipitation (ChIP)

The ChIP assay was conducted as previously described [25] using an anti‐p65 antibody and normal rabbit IgG. Immunoprecipitated DNA was purified and quantified by qPCR. Primers targeting the BNIP3L promoter and a negative control region in Exon 3 of BNIP3 were used, along with the NF‐κB binding motif in the IL6 gene promoter as a positive control [28] (Table S2).

### 2.12 Mass Spectrometry (MS)‐Based Proteomics Analysis

Proteins were isolated from cells and digested with trypsin. The peptides were purified using a C18 column and analysed on a Bruker timsTOF Pro mass spectrometer equipped with a CaptiveSpray ion source, coupled to a nanoElute liquid chromatography (LC) system. Data acquisition was performed in PASEF mode with 10 scans per topN cycle, excluding singly charged precursors. MS and MS/MS raw data were processed using Fragpipe software (v21.1) and searched against the human UNIPROT database containing 20,421 reviewed entries. Differentially expressed proteins were identified using *t*‐test analysis, with a fold‐change threshold of > 2 or < 0.5, with a significance cutoff (*p*‐value) of < 0.05.

### 2.13 Statistical Analysis

Descriptive statistics, that is, means ± SD, are shown on the figures. Two sample *t* tests were performed for data analysis. *p* < 0.05 was considered statistically significant. All tests were two‐sided.

## 3 Results

### 3.1 Ovarian CSCs Have Enhanced Mitophagy

**FIGURE 1:**
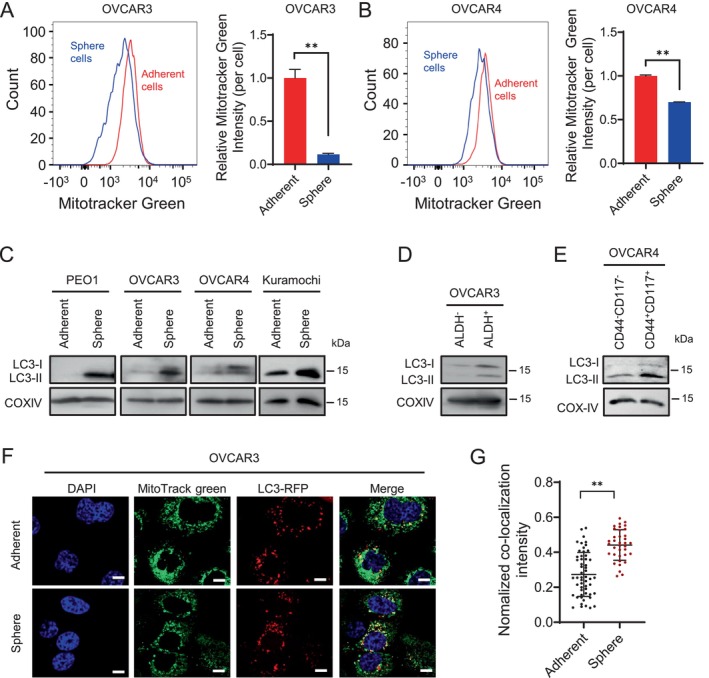
Ovarian CSCs exhibit enhanced mitophagy. (A, B) Mitochondrial mass was evaluated in spheroid and adherent cultured ovarian cancer cells. OVCAR3 and OVCAR4 cells cultured under serum‐free spheroid or serum‐containing adherent culture conditions were stained with MitoTracker Green and analysed using flow cytometry. *n* = 3, bar: SD, *p < 0.05, *p* < 0.01. (C–E) Mitochondria‐associated LC3 levels were assessed in ovarian CSCs. Mitochondria were isolated from a panel of spheroid and adherent cultured ovarian cancer cell lines (C), ALDH^+^ and ALDH^−^ cells sorted from the OVCAR3 cell line (D), and CD44^+^CD117^+^ and CD44^−^CD117^−^ cells sorted from the OVCAR4 cell line, immunoblotting was conducted to assess LC3 expression levels. (F, G) Confocal images of MitoTracker Green labelled mitochondria and of RFP‐labelled LC3 in adherent and spheroid cultured ovarian cancer cells. OVCAR3 cells were stably transfected with LC3‐RFP, cultured under either spheroid or adherent conditions. Cells were treated with Baf A1 for 18 h, followed by staining with MitoTracker Green to visualise mitochondria. RFP‐labelled LC3 and MitoTracker Green‐labelled mitochondria were examined using a confocal microscope. Scale bar: 10 μm (F). The intensity of colocalized LC3‐RFP was quantified and normalised to the average LC3‐RFP intensity in each cell (G). Bar: SD, *p* < 0.01.

CSCs have been reported to rely on mitophagy as a key survival mechanism to support their growth, propagation, and tumorigenic potential [29]. To investigate the role of mitophagy in ovarian CSCs, we enriched CSCs from HGSOC cell lines under spheroid culture conditions. The mitochondrial contents in both spheroid and adherent HGSOC cells were measured using the Mito Tracker Green staining and flow cytometry. We found a reduced amount of total mitochondria in spheroid cultured cells (Figure 1A,B), suggesting that ovarian CSCs may undergo enhanced mitophagy to remove damaged mitochondria.

LC3‐II binding to mitochondria is a hallmark of mitophagy. To further validate the elevated mitophagy activity in CSCs, we treated a panel of spheroid and adherent cultured HGSOC cell lines with Bafilomycin A1 (Baf A1) to accumulate LC3‐II associated mitochondria. Subsequently, mitochondria were isolated and analysed by immunoblotting to quantify mitochondria‐associated LC3‐II levels. Our results revealed that spheroid‐cultured HGSOC cells displayed higher mitochondrial LC3‐II expression compared to their adherent counterparts (Figure 1C), indicating enhanced mitophagy in ovarian CSCs. Elevated mitochondrial LC3‐II levels were also observed in CSCs characterised by high aldehyde dehydrogenase (ALDH) activity (ALDH+) (Figures 1D and S1A), and in CSCs identified by the CD44^+^CD117^+^ phenotype (Figures 1E and S1B).

To directly visualise mitophagy in adherent and spheroid ovarian cancer cells, we cultured OVCAR3 cells stably expressing RFP‐labelled LC3 under both conditions. Following treatment with Baf A1 to promote the accumulation of mitochondria undergoing mitophagy, cells were stained with MitoTracker Green to visualise mitochondria. Confocal imaging revealed that spheroid cells exhibited significantly greater colocalization of LC3 and mitochondria compared to adherent cells (Figure 1F,G). These findings collectively indicate that ovarian CSCs display elevated mitophagy relative to non‐CSC cells.

### 3.2 BNIP3 And BNIP3L Are Essential for Driving Enhanced Mitophagy in CSCs

**FIGURE 2:**
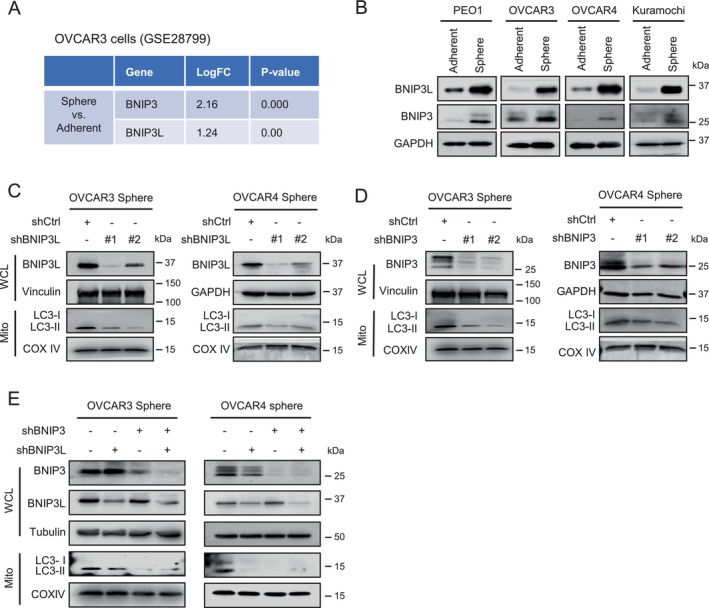
BNIP3L and BNIP3 are critical to the enhanced mitophagy in ovarian CSCs. (A) Differential expression of BNIP3 and BNIP3L in spheroid and adherent cultured OVCAR3 cells identified in dataset GSE28799. (B) BNIP3L and BNIP3 expression were assessed in a panel of adherent and spheroid cultured ovarian cancer cell lines. (C–E) Effect of BNIP3L or BNIP3 knockdown on mitophagy in spheroid cultured ovarian cancer cells. Spheroid cultured OVCAR3 and OVCAR4 cells were transfected with BNIP3L (C) or BNIP3 (D) shRNA individually or simultaneously (E). Whole cell lysates were analysed for BNIP3L and BNIP3 expression, with Vinculin, GAPDH, or Tubulin serving as a loading control. Mitochondria were isolated after cells were treated with Baf A1 for 18 h and LC3 levels were examined, with COX IV used as the mitochondrial loading control.

To determine the mitophagy pathways associated with ovarian CSCs, we conducted Gene Set Enrichment Analysis (GSEA) on transcriptomic data from OVCAR3 cells cultured under adherent and spheroid conditions. The analysis revealed a significant upregulation of BNIP3 (LogFC = 2.16) and BNIP3L (LogFC = 1.24) in spheroid cells compared to adherent cells (Figure 2A), suggesting that receptor‐mediated mitophagy may drive the enhanced mitophagy observed in ovarian CSCs. To validate this finding, we examined the protein and mRNA expression levels of BNIP3 and BNIP3L in adherent and spheroid‐cultured HGSOC cell lines. Consistently, both BNIP3 and BNIP3L were upregulated at the protein and mRNA levels in spheroid cultures across all tested cell lines (Figures 2B and S2A–D).

To determine the potential involvement of BNIP3 and BNIP3L in mitophagy in ovarian CSCs, we knocked down BNIP3 and BNIP3L individually and simultaneously in spheroid‐cultured OVCAR3 and OVCAR4 cells. Mitochondria‐associated LC3 levels were then analysed in the presence of Baf A1 to evaluate autophagic flux. We found that knockdown of either *BNIP3* or *BNIP3L* reduced mitochondria‐associated LC3 levels (Figure 2C,D), indicating that both proteins contribute to mitophagy in ovarian cancer stem cells (CSCs). However, simultaneous knocking down of *BNIP3* and *BNIP3L* did not further decrease LC3 levels compared to individual knockdowns, suggesting that they may regulate mitophagy through distinct, non‐redundant pathways.

### 3.3 BNIP3/BNIP3L Plays a Critical Role in Maintaining CSCs

**FIGURE 3:**
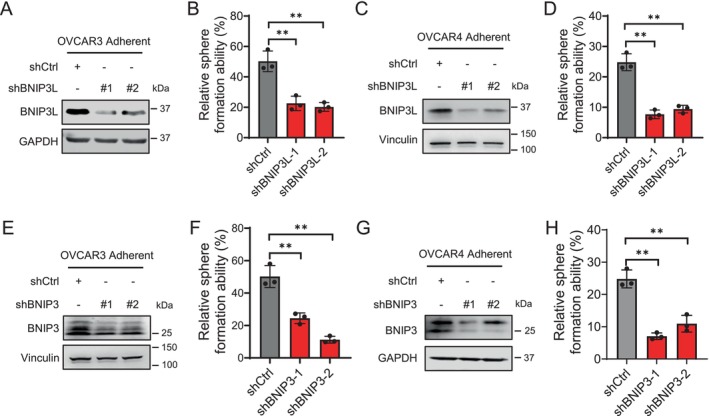
BNIP3L and BNIP3 play important roles in the maintenance of ovarian CSCs. (A–D) Effect of BNIP3L knockdown on the sphere formation ability. Adherent cultured OVCAR3 (A, B) and OVCAR4 (C, D) cells were transfected with BNIP3L shRNA (A, C), the sphere formation ability was assessed using the sphere formation assay (B, D). (E–H) Effect of BNIP3 knockdown on the sphere formation ability. Adherent cultured OVCAR3 (E, F) and OVCAR4 (G, H) cells were transfected with BNIP3 shRNA (E, G), the sphere formation ability was assessed using the sphere formation assay (F, H).

Having established the essential role of BNIP3 and BNIP3L in regulating mitophagy in ovarian CSCs, we next investigated whether BNIP3‐ and BNIP3L‐mediated mitophagy contributes to the maintenance of CSC stemness. To this end, we knocked down BNIP3L in adherent OVCAR3 and OVCAR4 cells and assessed their sphere‐forming ability, a characteristic of CSCs. Our results showed that BNIP3L knockdown significantly inhibited sphere formation capacity (Figures 3A–D and S3A). Similarly, BNIP3 knockdown also impaired sphere formation ability in both ovarian cancer cell lines (Figures 3E–H and S3B). Collectively, these findings suggest that BNIP3 and BNIP3L are critical for maintaining ovarian CSC stemness, probably through their role in promoting mitophagy.

### 3.4 NF‐κB Signalling Upregulates BNIP3L and BNIP3 Expression in Ovarian Cancer Cells

NF‐κB signalling has been implicated in the regulation of mitophagy [23], and is known to be highly activated in CSCs [30]. Notably, our analysis revealed that ovarian CSCs, identified by ALDH^+^, and enriched through spheroid culture, exhibit elevated NF‐κB signalling (Figure S4A,B). Furthermore, we observed increased nuclear accumulation of RelA/p65 in spheroid cultured cells compared to their adherent counterparts (Figure S4C,D), further supporting the notion that ovarian CSCs possess enhanced NF‐κB signalling.

Next, we examined the role of NF‐κB in regulating mitophagy in ovarian cancer cells and CSCs. We utilised OVCAR3 cells stably transfected with SparQ‐IKBSR, a cumate‐inducible constitutively active IκBα mutant that inhibits NF‐κB activation. Induction of SparQ‐IKBSR with cumate led to a reduction in mitochondria‐associated BNIP3L and LC3‐II levels (Figure S5A). In addition, NF‐κB inhibition attenuated TNF‐α‐induced mitophagy in adherent OVCAR3 cells (Figure S5B). Consistently, RelA/p65 knockdown in spheroid‐cultured OVCAR3 and OVCAR4 cells decreased mitochondria‐associated LC3‐II levels (Figure S5C,D). Moreover, pharmacological inhibition of NF‐κB signalling with BAY 11‐7082 and JSH‐23 similarly reduced mitochondria‐associated LC3‐II levels in spheroid OVCAR3 cells (Figure S5E,F). Collectively, these findings indicate that NF‐κB signalling plays a crucial role in promoting mitophagy in ovarian cancer cells and CSCs.

**FIGURE 4:**
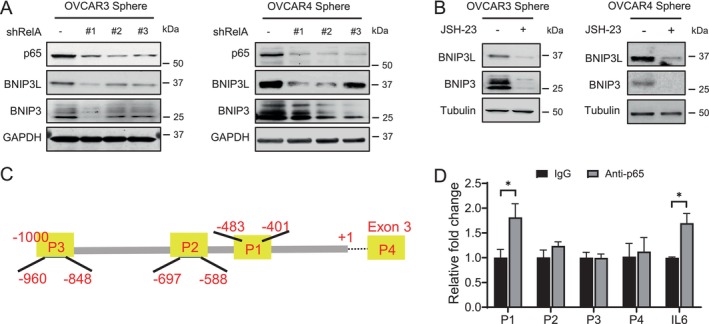
NF‐κB signalling upregulates expression of BNIP3L and BNIP3 in ovarian cancer cells. (A) Effect of RelA/p65 knockdown on BNIP3L and BNIP3 expression in ovarian cancer cells. Spheroid cultured OVCAR3 and OVCAR4 cells were transfected with RelA shRNA; BNIP3L and BNIP3 expression levels were assessed by Western blotting. (B) Effect of NF‐κB inhibitor on BNIP3L and BNIP3 expression in ovarian cancer cells. Spheroid cultured OVCAR3 and OVCAR4 cells were treated with JSH‐23 (10 μM) for 48 h; BNIP3L and BNIP3 expression levels were assessed by Western blotting. (C) The schematic diagram of putative RelA/p65 binding sites in the BNIP3L gene promoter (P1, P2, and P3) that are used for ChIP‐RT‐PCR analysis. P4 in Exon 3 serves as a negative control. (D) ChIP assay demonstrating the binding of RelA/p65 to the BNIP3L gene promoter. Chromatin from spheroid‐cultured OVCAR3 cell lysates was immunoprecipitated using an anti‐p65 antibody. RT‐PCR was conducted to quantify the enrichment of P1–P4 regions in the BNIP3L gene. The RelA/p65 binding site at the IL6 gene promoter was analysed as a positive control. *N* = 3, bar: SD, *p < 0.05.

Given that NF‐κB can regulate mitophagy‐related gene expression [23], we examined the effect of NF‐κB inhibition on BNIP3 and BNIP3L expression. Suppressing NF‐κB activity, either by RelA/p65 knockdown or treatment with the NF‐κB inhibitor JSH‐23, significantly reduced BNIP3 and BNIP3L levels in spheroid‐cultured OVCAR3 and OVCAR4 cells (Figure 4A,B). Previous studies have shown that RelA/p65 can bind to the BNIP3 promoter, functioning as a transcriptional repressor by antagonising E2F1 binding in normal cells [31]. However, its potential interaction with the BNIP3L promoter remains unclear. Using the UCSC Genome Browser, we identified three putative NF‐κB binding sites within the BNIP3L promoter sequence. To further investigate the potential interaction between NF‐κB and BNIP3L, we conducted chromatin immunoprecipitation (ChIP) using the anti‐p65 antibody in OVCAR3 sphere cells. The IL6 promoter was used as a positive control [28], whereas a sequence in Exon 3 of BNIP3L served as a negative control, both assessed by qPCR. Our results showed that anti‐p65 precipitated an increased amount of the P1 region in the BNIP3L gene (Figure 4C,D), suggesting a potential interaction between p65 and the BNIP3L promoter. Taken together, these results suggest that NF‐κB signalling upregulates both BNIP3 and BNIP3L expression, which may represent a key mechanism underlying NF‐κB‐mediated mitophagy.

### 3.5 Enhanced DNA‐PKs Contribute to NF‐κB‐Mediated Mitophagy

**FIGURE 5:**
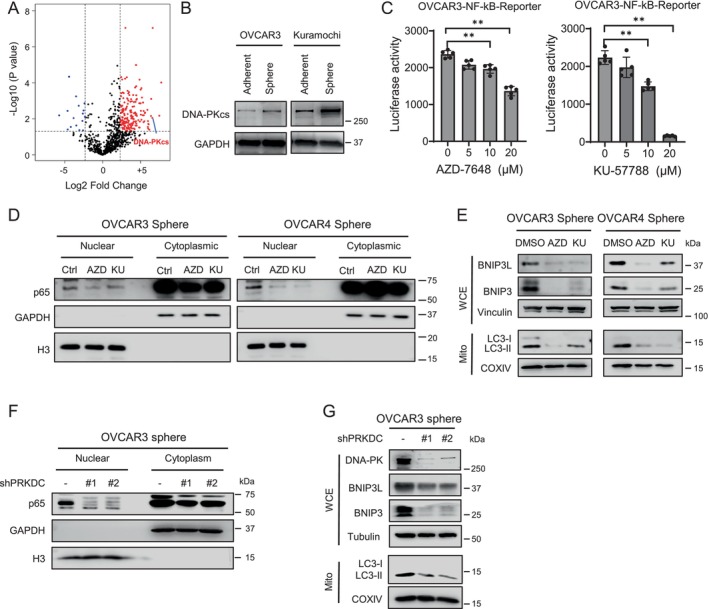
Increased expression of DNA‐PKcs contributes to NF‐κB‐mediated mitophagy in ovarian CSCs. (A) Differentially expressed proteins identified by LC–MS in spheroid and adherent cultured OVCAR3 cells. (B) Immunoblot analysis of DNA‐PKcs expression in adherent and spheroid cultured cells. (C, D) Effect of DNA‐PK inhibition on NF‐κB activity. OVCAR3 cells expressing an NF‐κB luciferase reporter were treated with DNA‐PK inhibitor AZD‐7648 or KU‐5778 for 48 h, followed by a luciferase assay to measure NF‐κB activity (C). Spheroid cultured OVCAR3 and OVCAR4 cells were treated with AZD‐7648 or KU‐5778 for 48 h, nuclei and cytoplasm were separated, and p65 expressed was determined (D). GAPDH serves as a cytoplasm loading control and Histone H3 (H3) as a nucleus loading control. (E) Effect of DNA‐PK inhibition on mitophagy in ovarian CSCs. Spheroid cultured OVCAR3 and OVCAR4 cells were treated with AZD‐7648 or KU‐5778 for 48 h, whole cell lysates were analysed for BNIP3L and BNIP3 expression, with Vinculin serving as a loading control. Mitochondria were isolated after cells were treated with Baf A1 for 18 h and LC3 levels was assessed, with COX IV serving as the mitochondrial loading control. (F, G) Effect of PRKDC knockdown on NF‐κB activity and mitophagy. Spheroid cultured OVCAR3 were transfected with PRKDC shRNA for 48 h, nuclei and cytoplasm were separated, and p65 expressed was determined (F). Whole cell lysates were analysed for DNA‐PKcs, BNIP3L and BNIP3 expression, with Tubulin as a loading control. Mitochondria were isolated after cells were treated with Baf A1 for 18 h and LC3 levels were assessed, with COX IV used as the mitochondrial loading control (G). *N* = 3, bar: SD, **p < 0.01.

NF‐κB signalling can be activated through multiple mechanisms, including DNA‐PK‐mediated p50 and NEMO phosphorylation [32, 33]. Notably, DNA‐PK catalytic subunit (DNA‐PKcs) has been reported to be highly expressed in glioma stem cells (GSCs) [34]. Our proteomic analysis comparing spheroid cultured and adherent cultured OVCAR3 cells similarly identified a significant upregulation of DNA‐PKcs (encoded by *PRKDC*) in spheroid cells (Figure 5A, Table S4). This finding was further confirmed in OVCAR3 and Kuramochi cells using immunoblotting (Figure 5B). To clarify the regulation of the NF‐κB pathway activity by DNA‐PK in ovarian cancer cells, we first treated OVCAR3 cells containing the NF‐κB reporter with two DNA‐PK inhibitors and assessed NF‐κB activity using the luciferase assay. As expected, treatment with DNA‐PKcs inhibitors KU‐67788 and AZD‐7648 significantly reduced NF‐κB activity in a dose–response manner (Figure 5C). We then treated spheroid cultured OVCAR3 and OVCAR4 cells with these inhibitors and demonstrated a reduced p65 nuclear localisation in these cells (Figure 5D). In addition, PRKDC knockdown using two distinct shRNAs similarly led to a substantial decrease in nuclear p65 levels (Figure 5F). All these results indicate that elevated DNA‐PKcs expression in ovarian CSCs plays a critical role in activating NF‐κB signalling.

Next, we evaluated mitophagy in spheroid cultured ovarian cancer cells following pharmacologic inhibition or genetic knockdown of DNA‐PKcs. Both DNA‐PK inhibitor treatment and DNA‐PKcs knockdown significantly reduced BNIP3L and BNIP3 expression, as well as decreased mitochondria‐associated LC3 levels (Figure 5E,G). These results indicate that the enhanced mitophagy observed in ovarian CSCs could be driven by elevated DNA‐PK expression, likely through DNA‐PK‐mediated NF‐κB activation, leading to the transcriptional upregulation of BNIP3L and BNIP3.

## 4 Discussion

Mitophagy plays a critical role in stem cell maintenance and plasticity. Studies have shown that PINK1‐dependent mitophagy influences the pluripotency and reprogramming efficiency of induced pluripotent stem cells (iPSCs) [35]. During the dedifferentiation of murine embryonic fibroblasts into iPSCs, BNIP3L expression is upregulated, leading to enhanced mitophagy, whereas BNIP3L silencing significantly reduces reprogramming efficiency [36]. Moreover, in vivo hyperactivation of PINK1/PARKIN‐dependent mitophagy expands the haematopoietic stem cells (HSCs) pool while reducing the population of differentiated blood cells [37], emphasising mitophagy's role in stemness maintenance. Similarly, enhanced mitophagy has been identified in CSCs and contributes to the maintenance of stemness in these cells [38]. This has been observed in various CSC types, including hepatic CSCs [7, 39], lung CSCs [40], oral CSCs [41], pancreatic CSCs [42], and ovarian CSCs [43]. In this study, we further demonstrate that ovarian CSCs enriched through serum‐free spheroid culture also exhibit elevated mitophagy; inhibition of BNIP3L/BNIP3‐mediated mitophagy impaired their self‐renewal ability. These findings highlight mitophagy as a key regulatory mechanism supporting stemness in ovarian CSCs.

Mitophagy regulation in CSCs varies across different types, involving distinct molecular pathways. Many CSCs exhibit heightened PINK1/Parkin activity, promoting mitochondrial quality control and survival. For instance, p52‐ZER6 enhances mitophagy via the PINK1/Parkin pathway in HCT116 stem‐like tumour spheres [44]; while ovarian CSCs derived from 3AO and SKOV3 cell lines through spheroid culture exhibit increased mitophagy via upregulated PINK1 [43]. Similarly, PINK1/Parkin‐mediated mitophagy is observed in hepatocellular carcinoma (HCC) CSCs [45]. In addition, receptor‐mediated mitophagy was also reported in CSCs, as hypoxia‐induced BNIP3 enhances mitophagy in oral squamous cell carcinoma CSCs [46]. In this study, we demonstrate that BNIP3 and BNIP3L are highly expressed in ovarian CSCs enriched from multiple cell lines via serum‐free spheroid culture, as well as in ALDH^+^ and CD44^+^CD117^+^ CSC populations. Knockdown of BNIP3 or BNIP3L reduces mitophagy and impairs self‐renewal. Since BNIP3 and BNIP3L have non‐redundant mitophagic roles in NK cells [47], our findings suggest that they cooperatively sustain elevated mitophagy and maintain CSC stemness in ovarian cancer.

BNIP3 and BNIP3L, primarily localising to the OMM, function as mitophagy receptors, facilitating the recruitment of autophagosomes to mitochondria [48, 49]. Their expression is frequently elevated in early‐stage human solid tumours, particularly under hypoxia [8, 50]. Hypoxic conditions stabilise HIF‐1α, which in turn induces BNIP3 and BNIP3L expression [51]. Beyond HIF‐1α, BNIP3L expression can also be upregulated by p53, SP‐1 [52], and NFE2L2/NRF2 [8], whereas BNIP3 expression is induced by RAS signalling [53] and E2F1 [54, 55], but repressed by NF‐κB via histone deacetylase‐1 (HDAC‐1) in rat ventricular myocytes [56, 57]. Here, we show that both BNIP3L and BNIP3 expression are upregulated by the hyperactivated NF‐κB signalling in ovarian CSCs, revealing a novel regulatory mechanism linking NF‐κB activation to mitophagy and CSC maintenance. We present evidence that RelA/p65 binds to the promoter region of the BNIP3L gene, suggesting this interaction as the likely mechanism through which NF‐κB activates BNIP3L transcription in ovarian CSCs. Although NF‐κB has been reported to repress BNIP3 transcription in rat ventricular myocytes by competing with E2F1 for promoter binding, it was nonetheless reported to bind the BNIP3 promoter region [56]. Based on our findings, we propose that in ovarian CSCs, NF‐κB enhances BNIP3 expression through direct promoter binding and transcriptional activition. Together, these results highlight a cell type–dependent regulatory mechanism by which NF‐κB modulates the expression of mitophagy‐related genes.

NF‐κB signalling is well established in promoting cellular survival and stress adaptation, hyperactivated NF‐κB also plays a crucial role in both enhancing mitophagy and sustaining the stemness properties of CSCs [23, 30]. Previously, NF‐κB has been shown to facilitate mitophagy by upregulating the expression of the p62/SQSTM1 adaptor protein, which mediates the removal of damaged mitochondria in macrophage to suppress inflammasome activation [23]. Here, we identified an additional mechanism through which NF‐κB promotes mitophagy. Given that knockdown of BNIP3 or BNIP3L compromised the self‐renewal capacity in ovarian cancer cells, our findings suggest that NF‐κB‐driven mitophagy contributes to CSC maintenance by enhancing their resilience to environmental stressors, including hypoxia and chemotherapeutic agents.

NF‐κB signalling is often constitutively activated in CSCs from various cancers [30]. Although multiple mechanisms have been proposed to explain the elevated NF‐κB activity in CSCs, the precise mechanisms driving its constitutive activation remain unclear. DNA‐PK, as a serine–threonine protein kinase, that is, mainly known as a critical component in the DNA damage response [58], has been reported to promote NF‐κB activation, mainly by phosphorylating p50 and NEMO [32, 33]. The catalytic subunit of DNA‐PK, DNA‐PKcs (encoded by *PRKDC*) was found to be highly expressed in glioma stem cells and contribute to the maintenance of GSCs by phosphorylating and stabilising Sox2 [34]. In this study, we also revealed upregulated DNA‐PKcs in ovarian CSCs. Genetic knockdown or pharmacological inhibition of DNA‐PKcs led to a reduction in NF‐κB activity, indicating that upregulated DNA‐PKcs is a contributor to the constitutively elevated NF‐κB activity in ovarian CSCs. Notably, DNA‐PKcs inhibition also compromised mitophagy in ovarian CSCs, suggesting that enhanced mitophagy in ovarian CSCs might be regulated through the DNA‐PKcs–NF‐κB axis. These findings highlight a potential link between DNA damage response signalling, NF‐κB activation, and mitochondrial quality control in ovarian CSCs.

**FIGURE 6:**
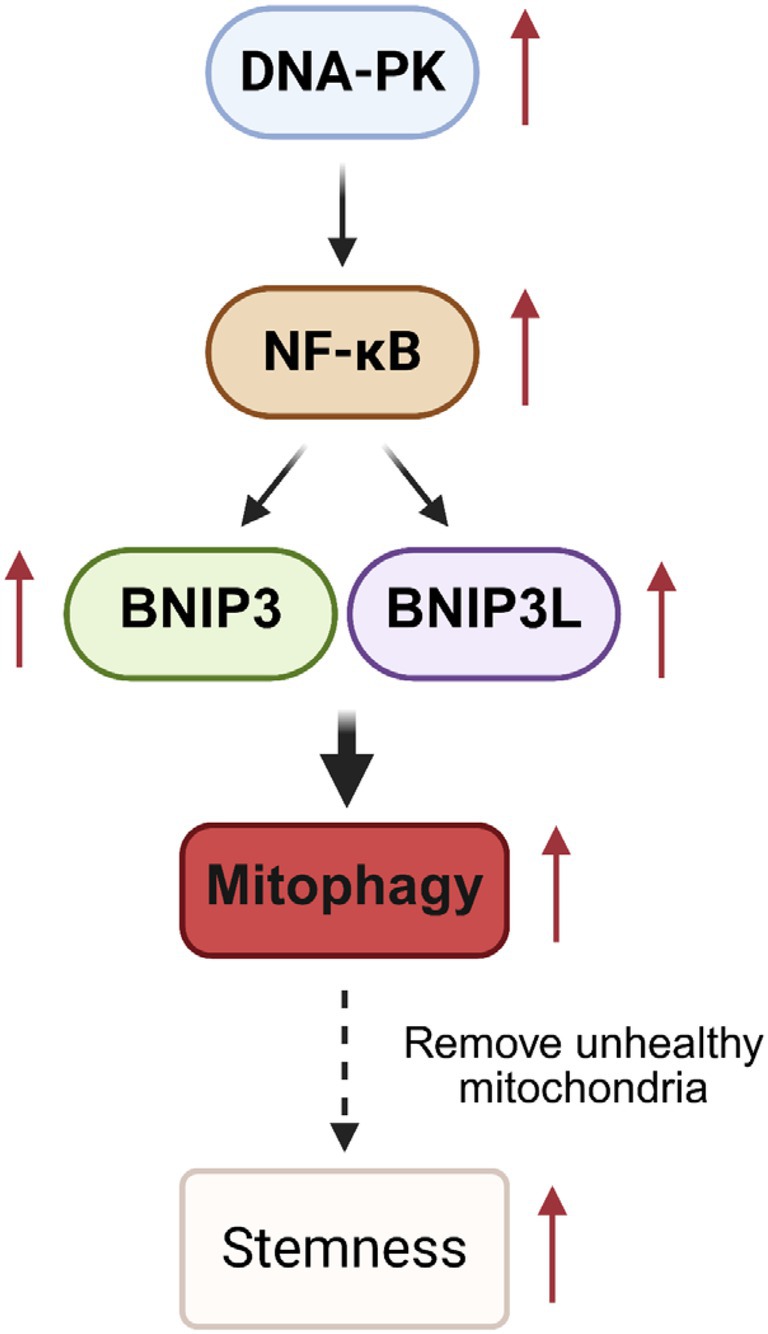
Schematic illustration of the DNA‐PK/NF‐κB/BNIP3L‐BNIP3 axis in regulating mitophagy in ovarian CSCs. Elevated DNA‐PKcs in ovarian CSCs enhances canonical NF‐κB signalling, which further increases the expression of BNIP3L and BNIP3, triggering receptor‐mediated mitophagy.

In summary, this study reveals novel insights into the regulation and function of mitophagy in ovarian CSCs. By elucidating the interplay between DNA‐PK‐mediated NF‐κB activation and mitophagy, our findings establish a mechanistic basis for targeting CSCs in ovarian cancer (Figure 6). Inhibiting the DNA‐PK/NF‐κB/BNIP3L‐BNIP3 axis presents a potential therapeutic strategy to disrupt CSC survival and overcome therapy resistance. These discoveries may contribute to the development of more effective treatment approaches, ultimately enhancing patient outcomes.

## Author Contributions

**Na Li:** conceptualization (equal), investigation (lead), methodology (equal), writing – original draft (lead). **Tejinder Pal khaket:** conceptualization (equal). **Yajing Yang:** conceptualization (equal). **Linzhou Wang:** conceptualization (equal). **Shurui Cai:** conceptualization (equal). **Aidan Li:** conceptualization (equal). **Elsa Wani:** conceptualization (equal). **Jessica Miao:** conceptualization (equal). **Nan Zhang:** conceptualization (equal). **Qingfei Zheng:** conceptualization (equal). **Junran Zhang:** conceptualization (equal). **Xuefeng Liu:** conceptualization (equal). **Selvendiran Karuppaiyah:** conceptualization (equal). **Dehua Pei:** conceptualization (equal). **Qi‐En Wang:** conceptualization (lead), data curation (equal), formal analysis (equal), funding acquisition (equal), investigation (equal), methodology (equal), supervision (lead), writing – original draft (lead), writing – review and editing (lead).

## Conflicts of Interest

The authors declare no conflicts of interest.

## Supporting information


**Figure S1.** Isolation of CSCs from ovarian cancer cell lines. A. Isolation of ALDH+.
**Figure S2.** Ovarian CSCs possess increased expression levels of BNIP3L and BNIP3.
**Figure S3.** BNIP3/BNIP3L are critical to the sphere formation ability of ovarian cancer cells.
**Figure S4.** Ovarian CSCs possess enhanced NF‐κB signalling. A, B. NF‐κB activity was assessed.
**Figure S5.** NF‐κB signalling upregulates mitophagy in ovarian cancer. A. OVCAR3 cells.

**Table S1.** Plasmids used in the study.
**Table S2.** Sequence of primers used in qRT‐PCR and ChIP‐PCR.
**Table S3.** Antibodies used in immunoblotting and FACS.

**Table S4.** Normalized Proteomics data.

## Data Availability

Data available in article Supporting Information.
