# Supplementary material for: BNIP3L/BNIP3‐Mediated Mitophagy Contributes to the Maintenance of Ovarian Cancer Stem Cells
Source: J Cell Mol Med. 2025 Oct 13;29(19):e70704. doi: 10.1111/jcmm.70704 (PMC12516242; doi:10.1111/jcmm.70704)
Supplement: Supplementary file 1 — Figure S1. Isolation of CSCs from ovarian cancer cell lines. A. Isolation of ALDH+. Figure S2. Ovarian CSCs possess increased expression levels of BNIP3L and BNIP3. Figure S3. BNIP3/BNIP3L are critical to the sphere formation ability of ovarian cancer cells. Figure S4. Ovarian CSCs possess enhanced NF‐κB signalling. A, B. NF‐κB activity was assessed. Figure S5. NF‐κB signalling upregulates mitophagy in ovarian cancer. A. OVCAR3 cells. [file JCMM-29-e70704-s003.pdf]

Supplementary Figures

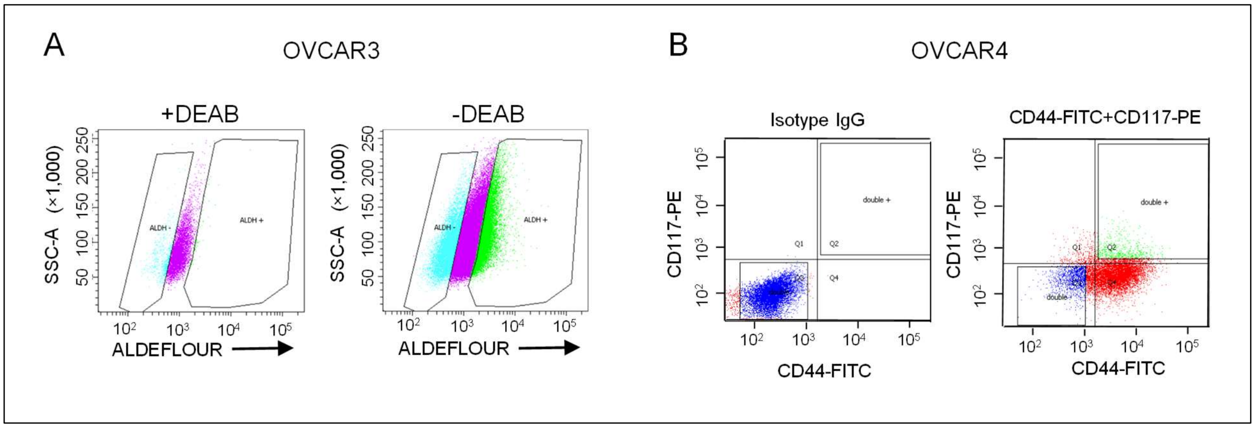

Fig. S1. Isolation of CSCs from ovarian cancer cell lines. A. Isolation of ALDH$^+$ and ALDH$^-$ cells. OVCAR3 cells were stained with ALDEFLUOR. DEAB was added to set up the gate. ALDH$^+$ and ALDH$^-$ cells were sorted using FACS. **B.** Isolation of CD44$^+$CD117$^+$ and CD44$^-$CD117$^-$ cells. OVCAR4 cells were incubated with FITC-conjugated anti-CD44 and PE-conjugated CD117 antibodies, CD44$^+$CD117$^+$ and CD44$^-$CD117$^-$ cells were sorted using FACS.

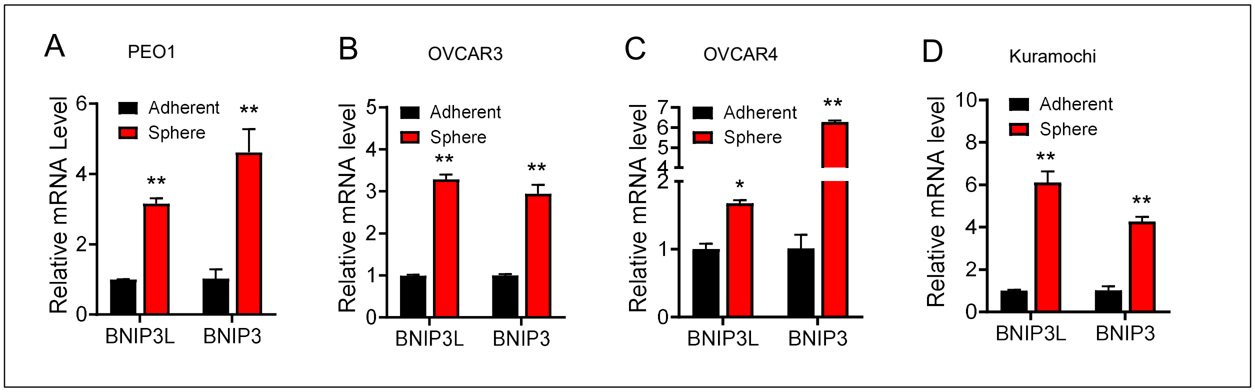

Fig. S2. Ovarian CSCs possess increased expression levels of BNIP3L and BNIP3. A panel of ovarian cancer cell lines PEO1 (A), OVCAR3 (B), OVCAR4 (C), Kuramochi (D) were cultured under either adherent or spheroid conditions. qRT-PCR was performed to assess the mRNA levels of BNIP3L and BNIP3. n = 3, bar: SD, *: $P < 0.05$; **: $P < 0.01$ compared to the corresponding adherent cultured cells.

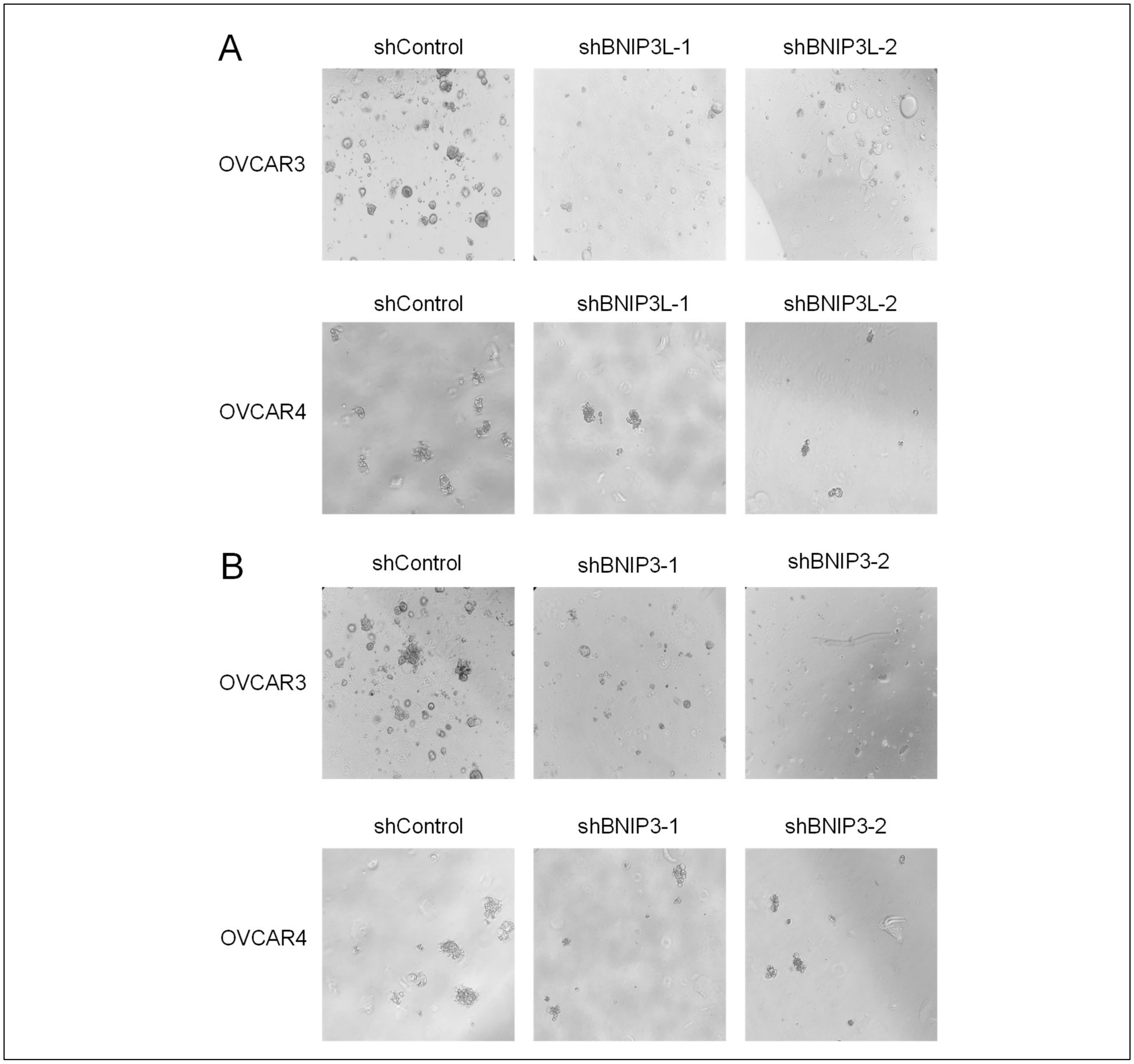

Fig. S3. BNIP3/BNIP3L are critical to the sphere formation ability of ovarian cancer cells. OVCAR3 and OVCAR4 cells were transfected with BNIP3L shRNA (A) or BNIP3 shRNA (B). 500 cells were seeded in semisolid media containing serum-free PromoCell 3D Tumor Sphere Medium XF in Ultra-Low Attachment plates and allowed to grow for 12 days. Representative images of colonies were shown.

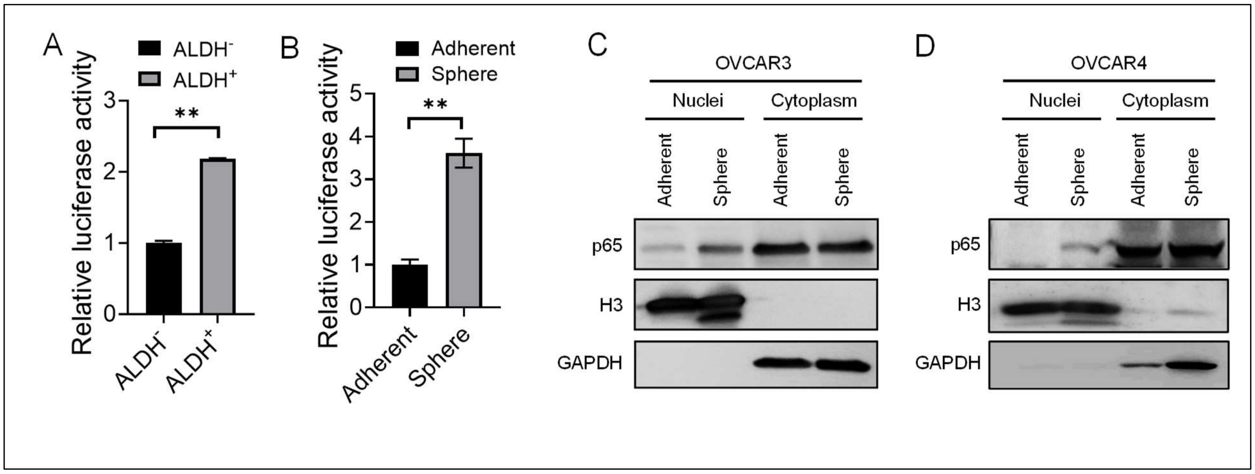

Fig. S4. Ovarian CSCs possess enhanced NF-κB signaling. A, B. NF-κB activity was assessed using the luciferase assay in $ALDH^+$ and $ALDH^-$ cells sorted from OVCAR3 cells transfected with NF-κB luciferase reporter (A), as well as spheroid and adherent cultured OVCAR3 cells transfected with NF-κB luciferase reporter (B). N = 3, bar: SD, **: $P < 0.01$. **C, D.** Adherent and spheroid cultured OVCAR3 (C) and OVCAR4 (D) were subjected to cellular fractionation to isolate nuclear and cytoplasmic protein fractions. Western blot analysis was conducted to determine p65 protein levels. Histone H3 (H3) and GAPDH were blotted to serve as nuclear and cytoplasmic loading controls, respectively.

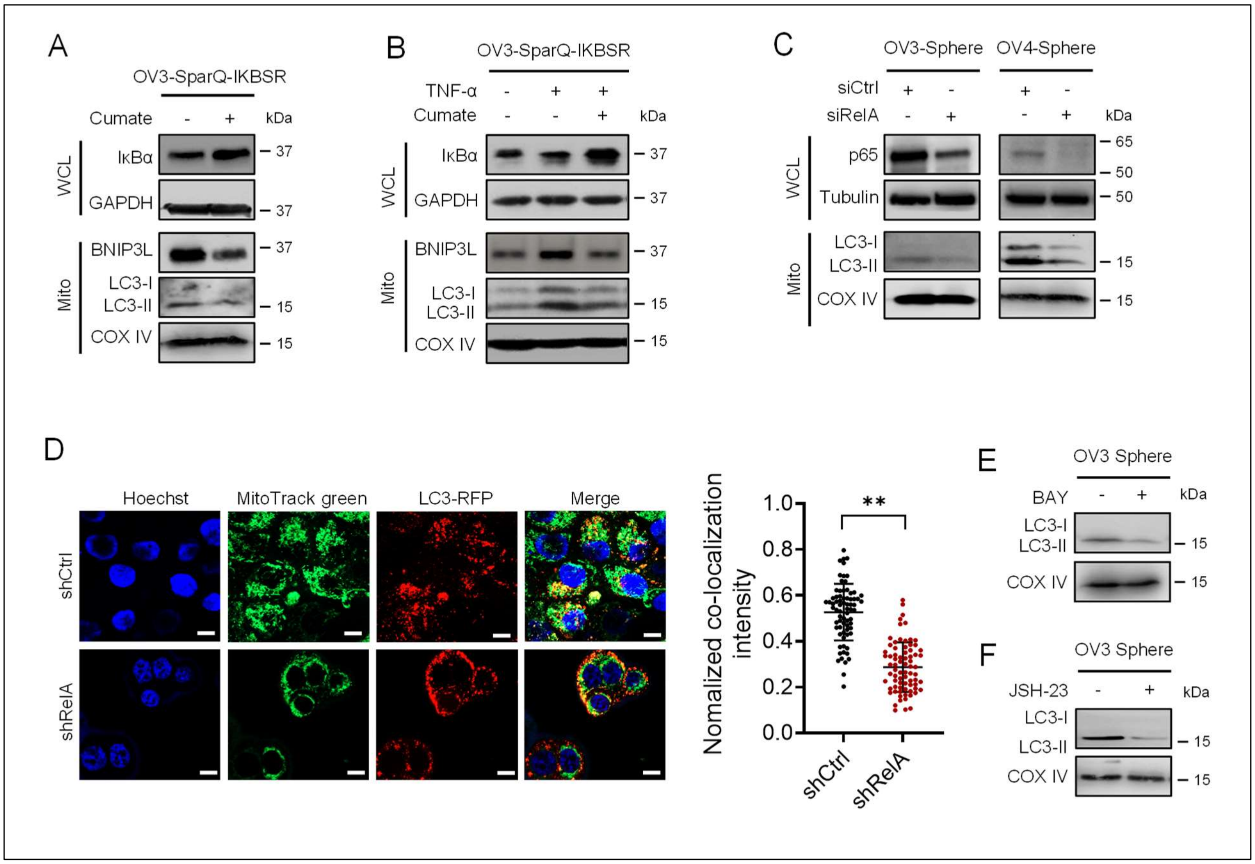

Fig. S5. NF-κB signaling upregulates mitophagy in ovarian cancer. A. OVCAR3 cells possessing cumate-inducible IKBSR expression vectors (OV3-SparQ-IKBSR) were treated with or without cumate for 3 days to induce IκB constitutive expression. Whole cell lysates were analyzed for IκB expression, with GAPDH serving as a loading control. Mitochondria were isolated and BNIP3L and LC3 levels was assessed, with COXIV serving as the mitochondrial loading control. **B.** OV3-SparQ-IKBSR cells were treated with TNF-α for 6 h in the absence of presence of cumate. Whole cell lysates were analyzed for IκB expression. Mitochondria were isolated and BNIP3L and LC3 levels was assessed. **C.** Spheroid cultured OVCAR3 and OVCAR4 cells were transfected with RelA/p65 siRNA or control siRNA for 48 hours, Whole cell lysates were analyzed for p65 expression. Mitochondria were isolated and LC3 levels was assessed. **D.** Spheroid cultured OVCAR3 cells with LC3-RFP expression, were further transfected with RelA or control shRNA. Mitochondria were stained with MitoTracker Green after incubation in the presence of Raf A1 for 18 h. RFP-labeled LC3 and MitoTracker Green-labeled mitochondria were examined using a confocal microscope. The intensity of colocalized LC3-RFP was quantified and normalized to the average LC3-RFP intensity in each cell. Bar: SD, **: $P < 0.01$ **E, F.** Spheroid cultured OVCAR3 cells were treated with NF-κB inhibitor BAY 11-7082 (10 μM) (E) and JSH-23 (10 μM) (F) for 48 h. Mitochondria were isolated and subjected to immunoblotting to assess the LC3 levels.
