# Supplementary material for: BNIP3L/BNIP3‐Mediated Mitophagy Contributes to the Maintenance of Ovarian Cancer Stem Cells
Source: J Cell Mol Med. 2025 Oct 13;29(19):e70704. doi: 10.1111/jcmm.70704 (PMC12516242; doi:10.1111/jcmm.70704)
Supplement: Supplementary file 2 — Table S1. Plasmids used in the study. Table S2. Sequence of primers used in qRT‐PCR and ChIP‐PCR. Table S3. Antibodies used in immunoblotting and FACS. [file JCMM-29-e70704-s002.pdf]

Supplementary Table S1-S3

Table S1: Plasmids used in the study

Plasmid	Sources	Identifier	Sequencing
shBNIP3-#1	Millipore Sigma	TRCN0000007830	GCCACGTCACTTGTGTTTATT
shBNIP3-#2	Millipore Sigma	TRCN0000007831	GCCTCGGTTTCTATTTATAAT
shBNIP3L-#1	Millipore Sigma	TRCN0000007847	CAGTCAGAAGAAGAAGTTGTA
shBNIP3L-#2	Millipore Sigma	TRCN0000007846	GCTAGGCATCTATATTGGAAA
shRelA-#1	Millipore Sigma	TRCN0000014687	CCTGAGGCTATAACTCGCCTA
shRelA-#2	Millipore Sigma	TRCN0000014684	CGGATTGAGGAGAAACGTAAA
shRelA-#3	Millipore Sigma	TRCN0000014683	GCCTTAATAGTAGGGTAAGTT
shPRKDC-#1	Millipore Sigma	TRCN0000194811	CAGTATTGAATTTCGTGAATG
shPRKDC-#2	Millipore Sigma	TRCN0000197152	GAAACAGCTGTCTCCGTAAAT
pmRFP-LC3	Addgene	21075	
pHAGE NFkB-TA-LUC-UBC-GFP-W	Addgene	49343	
siRELA	Invitrogen	S11915	

Table S2: Sequence of primers used in qRT-PCR and ChIP-PCR

Primers	Forward (5'-3')	Reverse (5'-3')
BNIP3	TGGACGGAGTAGCTCCAAGA	CCACCCCAGGATCTAACAGC
BNIP3L	GAACAGCAGCAATGGCAATGA	GCCCATCTTCTTGTGGCGAA
P1	GCACCTGGGTTTGGGGATTC	GCACGCATCTGTTTTCTACCTC
P2	TGGGATTTGGGTGAGTACTTTCT	TCGATTACAAACACACCATCATTCT
P3	CTTGGTGTGACTGTCTCCCC	TCGCAGTCTCTAACTTGTCTTTTAATT
P4	TCGTGTTTGCCTGTAGCTGA	TTTTCTTTTCTCTCCCACGGGT
IL6	TTCCAATCAGCCCCACCCG	CTCCAATCCTATATTTATTGGGGGT

Table S3: Antibodies used in immunoblotting and FACS

Antibodies	Sources	Identifier	Dilution
Rabbit monoclonal Anti-BNIP3	Cell Signaling Technology	44060S	1:1000 WB
Rabbit monoclonal Anti-BNIP3L	Cell Signaling Technology	12396S	1:1000 WB
Rabbit monoclonal Anti-LC3B	Cell Signaling Technology	3868S	1:1000 WB
Mouse monoclonal Anti-COXIV	Cell Signaling Technology	11967S	1:1000 WB
Mouse monoclonal Anti-IKBa	Cell Signaling Technology	4814S	1:2000 WB
Rabbit monoclonal Anti-Histone H3	Cell Signaling Technology	4499S	1:3000 WB
Rabbit Polyclonal Anti-DNA-PKcs	Proteintech	19983-1-AP	1:1000 WB
Rabbit Polyclonal Anti-RelA/p65	Thermo Fisher	PA5-16545	1:1000 WB<br>1:50 ChIP
Mouse monoclonal Anti-GAPDH	Santa Cruz Biotechnology	SC-365062	1:5000 WB
Mouse monoclonal Anti-Tubulin	Cell Signaling Technology	3873S	1:5000 WB
Mouse monoclonal Anti-Vinculin	Santa Cruz Biotechnology	sc-73614	1:5000 WB
Goat Anti-Rabbit IgG Antibody, HRP conjugate	Sigma	12-348	1:5000 WB
Goat Anti-Mouse IgG Antibody, HRP conjugate	Sigma	12-349	1:5000 WB
PE-conjugated CD117 antibody	BD Pharmingen	555714	FACS
FITC-conjugated CD44 antibody	BD Pharmingen	555478	FACS
